# Cincinnati Medical School

**Published:** 1838

**Authors:** 


### III.—ORIGINAL.

### THE WESTERN JOURNAL.

*E Sylvis Nuncius.*

CINCINNATI, OHIO, JULY, 1838.

### Art. I.—The Cincinnati College.

If we frequently refer to this institution it is because our Journal is its advocate, and for the additional reason, that the newspaper press of the city has been, and still remains, nearly silent in reference to it. Out of twelve or fourteen papers, not more than two or three have ventured to notice either it or our Journal. With the reasons for their silence we have no concern. We shall not, indeed, offer any explanation of the phenomena, that our distant readers, may throughout the year, take up a Cincinnati newspaper, and read it from alpha to omega, without discovering, that a medical institution, more comprehensive in its plan, with a greater number of professors, and a longer session, than most of the schools in the United States, was organized in 1835; and has been prosecuted, successfully, through three sessions, with classes of 66, 85 and 125; and still such is the fact. It is further true, that the class of last year, was composed of young gentlemen from Ohio, Kentucky, Tennessee, Alabama, Mississippi, Missouri, Illinois, Indiana, Pennsylvania, Virginia, North Carolina, New York and New England; and that the distant state of Alabama gave us eighteen, a greater number, we believe, than she gave to any other institution in the Union. Such a progress, in no manner or degree the result of any extraneous or temporary influence, has convinced us, that we have been prosecuting a *good* object in the *right* way, that is, by means of hard and unremitting labour. We began, like younger sons, without endowment or heritage of any kind; and had, moreover, to grapple with the opposition of two pampered institutions in the West, both of whom declined to acknowledge us:—one of them even refusing officially to correspond with us on any subject; and the other not consenting to admit a student from our school to the *ad eundum* in theirs, altho' we had a greater number of professors, taught more branches, and had a longer session than they ! Such hostility would have quite discouraged us, had we not stumbled upon the theory, that their apparent contempt, was, in fact, the manifestation of fear. This theory proved to be correct, for our second session disorganized both institutions; and promoted the establishment of a new one, out of the scattered fragments. In that session, ours was in point of number, the third school, out of three, in the Valley of the Ohio; but in the next session, that of 1837—8, it was the second out of four. These are facts, and we give publicity to them, believing that we are entitled to all the benefit they can bring us. They are the fruits of honest enterprise, and we do not feel, that it is boastful thus to put them forth; at all events, should criticism denounce them as vanity, we wish it known, that the individual who is writing this article, and he only, is justly obnoxious to her shafts. His colleagues do not participate in the authorship, and must not, therefore, be involved in the consequences. Having, thus "assumed the responsibility," we shall continue our narrative. We repeat, then, that our medical institution is, emphatically, the offspring of our own enterprise. That we have repaired a decayed edifice, from whose walls nearly every thing had *sloughed* off—that we have three elegible lecture-rooms, each capable of accommodating 300 students—a room for practical anatomy, 30 by 45 feet with every requisite convenience—a museum of healthy and morbid anatomy—a laboratory of chemical apparatus—a library of 1300 volumes, and a hospital contiguous to the College edifice. In the collection and creation of these means of instruction, we have been aided to a moderate extent by our fellow citizens, but the professors themselves have expended upwards of *twelve thousand dollars*, a larger sum, if we mistake not, than has yet been disbursed by any Faculty of professors of the United States, in founding an institution.

Thus we have put into our own hands the means of instruction, and shall not wield them the less effectually, *because* they are of our own creation. Such physicians then as may choose to advise their pupils to try us, will find, whatever may be our defects, that a glowing and inextinguishable zeal, animates the heart of every professor.

We have intimated that the two schools, existing in the West, when ours was organized, refused to acknowledge us. We should add, that the one established in this city, has had an opportunity, practically, to receive but one pupil of ours for a second course; while 22 of its have been graduated by us; and that the school at Lexington notwithstanding her refusal to recognize us, has sent her circulars to our pupils, informing them, that if they would come to that University, they should have credit for the course of lectures they had attended here. We have not, however, known of more than one or two who availed themselves of the permission thus insidiously given; whle we have already graduated ten students who had attended their first course in that establishment. As to the institutions east of the Mountains, we can state, that letters recognizing us, have been received from them, generally, from New-England to Carolina. For the terms of tuition, length of session and other details, we refer to the circular of the Dean on the left page of this number.

Such is the present condition of the Medical Department of the Cincinnati College. It is no longer an experiment. It possesses every requisite, (except genius and learning in its professors) and these can at any time be bestowed on them by a circular of the Board of Trustees. One of the functions of every literary confederation is that of conferring these high attributes—of raising the ordinary into extraordinary, by that most convenient lever, the pen. If *our* Trustees do not annually exercise this corporate franchise, and renovate our intellectual constitution, it is their own fault. They published originally, that we "are professors in whom they have confidence," and there they stopped. They courageously and composedly left the rest to ourselves. We were touched by the compliment, and have laboured diligently to show ourselves worthy of it. We have not importuned them to send forth either eulogiums or biographical sketches—we have not asked to be wrapped up, like nostrums, in certificates, stamped with the seal of the corporation. But if we have not prayed to Hercules, we have placed our shoulders to the wheels, and there they will remain, till we demonstrate the truth of our motto—LABOR VINCIT OMNIA. D.

